# Reducing Flower Competition for Assimilates by Half Results in Higher Yield of *Fagopyrum esculentum*

**DOI:** 10.3390/ijms21238953

**Published:** 2020-11-25

**Authors:** Marta Hornyák, Aneta Słomka, Klaudia Sychta, Michał Dziurka, Przemysław Kopeć, Jakub Pastuszak, Anna Szczerba, Agnieszka Płażek

**Affiliations:** 1 Department of Plant Physiology, Faculty of Agriculture and Economics, University of Agriculture, Podłużna 3, 30-239 Kraków, Poland; marta.golebiewska1990@gmail.com (M.H.); kubapaaa@gmail.com (J.P.); anna.szczerba21@gmail.com (A.S.); rrplazek@cyf-kr.edu.pl (A.P.); 2 Department of Plant Cytology and Embryology, Institute of Botany, Faculty of Biology, Jagiellonian University in Kraków, Gronostajowa 9, 30-387 Kraków, Poland; klaudia.sychta@uj.edu.pl; 3 F. Górski Institute of Plant Physiology, Polish Academy of Sciences, Niezapominajek 21, 30-239 Kraków, Poland; m.dziurka@ifr-pan.edu.pl (M.D.); przemyslawkopec@gmail.com (P.K.)

**Keywords:** common buckwheat, embryo sacs, nutrient stress, phytohormones, pollen grains, yield parameters

## Abstract

Despite abundant flowering throughout the season, common buckwheat develops a very low number of kernels probably due to competition for assimilates. We hypothesized that plants with a shorter flowering period may give a higher seed yield. To verify the hypothesis, we studied nutrient stress in vitro and in planta and analyzed different embryological and yield parameters, including hormone profile in the flowers. In vitro cultivated flowers on media with strongly reduced nutrient content demonstrated a drastic increase in degenerated embryo sacs. In in planta experiments, where 50% or 75% of flowers or all lateral ramifications were removed, the reduction of the flower competition by half turned out to be the most promising treatment for improving yield. This treatment increased the frequency of properly developed embryo sacs, the average number of mature seeds per plant, and their mass. Strong seed compensation under 50% inflorescence removal could result from increased production of salicylic and jasmonic acid that both favor more effective pollinator attraction. Plants in single-shoot cultivation finished their vegetation earlier, and they demonstrated greater single seed mass per plant than in control. This result suggests that plants of common buckwheat with shorter blooming period could deliver higher seed yield.

## 1. Introduction

Common buckwheat (*Fagopyrum esculentum* Moench) is a valuable plant of *Polygonaceae* family, grown mainly for human food due to favorable chemical composition of its fruits (commonly termed seeds), especially substantial content of lysine and other amino acids. Seeds are also gluten free [1]. Nectar of common buckwheat is a source of valued honey, while grain and straw are used as animal feed. The presence of two morphs of flower, Pin and Thrum, is a manifestation of heteromorphic self-incompatibility (heterostyly). Both types of flowers exhibit differences in pistil and stamen lengths [2]. Fertilization occurs only following cross-pollination between two different types of flowers.

Factors such as low resistance to excessive water, lodging, and pre-harvest sprouting occurring before flowering affect buckwheat yield, which is low and variable [3].

Plant yield is also affected by the short life span (1–2 days) of individual flowers and disturbances in female but not in male line development. The number of degenerated ovule sacs in plants is high and ranges from 10% to 30%, depending on the strain and cultivar [4]. Additionally, buckwheat flowering is sensitive to many environmental stresses, including frost, heat, and drought occurring in the spring and summer despite stronger vegetative growth at 30 °C than at 20 °C [5]. A drastic reduction in the number of properly developed embryo sacs was clear in open flowers at 30 °C in cultivars ‘Panda’ and ‘Korona’ (= strain PA15) [5,6]. Similarly, sensitivity to the thermal stress was shown by Slawinska and Obendorf [1]; plants grown at 18 °C had by 40% increased seed set. They set seeds over a longer period and produced by 40% more dry mass per seed than plants grown at 25 °C. Several authors [7,8,9] described ultrastructural changes in reproductive cells under thermal stress and suggested that premature synergid collapse may prevent a pollen tube from reaching the embryo sac. It was recently shown that radiation restriction resulting from plant growth could have increased floret mortality and thereby decrease the number of achenes (fruits) per raceme (type of inflorescence in common buckwheat) [10]. High temperature and other environmental factors cause premature flower and immature seed fall. A considerable increase in abscisic acid in open flowers ready for fertilization under thermal stress may serve as a signal inducing flower senescence observed in the next few days [6]. The results of a project carried out during 2014–2016 (‘Increasing the vitality and degree of pollination of buckwheat flowers in order to obtain a higher seed yield’), concerning flowering and yielding biology of Polish buckwheat accessions, indicate that the more flowers a plant produces, the greater their abortion, and the number of flowers negatively correlates with the number of mature seeds. Therefore, increasing the number of flowers per plant may not improve the yield It is, however, opposite to a selection index, which was constructed based on seven agro-morphological traits measurable in a single plant, showing that seed yield is positively correlated with the number of flower clusters in common buckwheat [11].

The frequency of aborted embryos in controlled conditions after hand-pollination is rather low (<10%) [12] or moderate (9.6–19.1%) [2,7,8,9], depending on the genotype, thus it seems that selective embryo abortion resulting from better or worse embryo fitness is not the case of low yield in common buckwheat. Halbrecq et al. [13] and Cawoy et al. [2] suggested that embryo abortion in common buckwheat is fixed by an internal mechanism at a relatively constant level and is not the result of insufficient nutrient supply from leaves. In contrast, studies in four Polish cultivars based on observation of embryo development with free access of pollinators showed that more, up to 28%, of embryos could have degenerated, some of them showing a typical hallmark of starvation [4]. This is in accordance with earlier observations of Inoue and Hagiwara [14] and Inoue et al. [15], who proposed that the percentages of flower fertilization and seed set are influenced by the degree of competition for nutrients between source and sink organs. Buckwheat blooms throughout the whole growing season, which may result in a strong competition for assimilates between the already set seeds and flowers still in production. To resolve these inconsistencies in the above-mentioned results, we analyzed various reproductive and yield parameters and the hormone profile of the flowers of cv. ‘Panda’ and ‘Korona’ of *F. esculentum* under in vitro and in planta conditions. Our aim was to investigate whether the seed limitation is associated with a strong sink restriction and linked to internal flower structure and fertilization. We hypothesized that plants with a shorter flowering period may give a higher seed yield.

## 2. Results

### 2.1. Embryological Analysis

**Figure 1:**
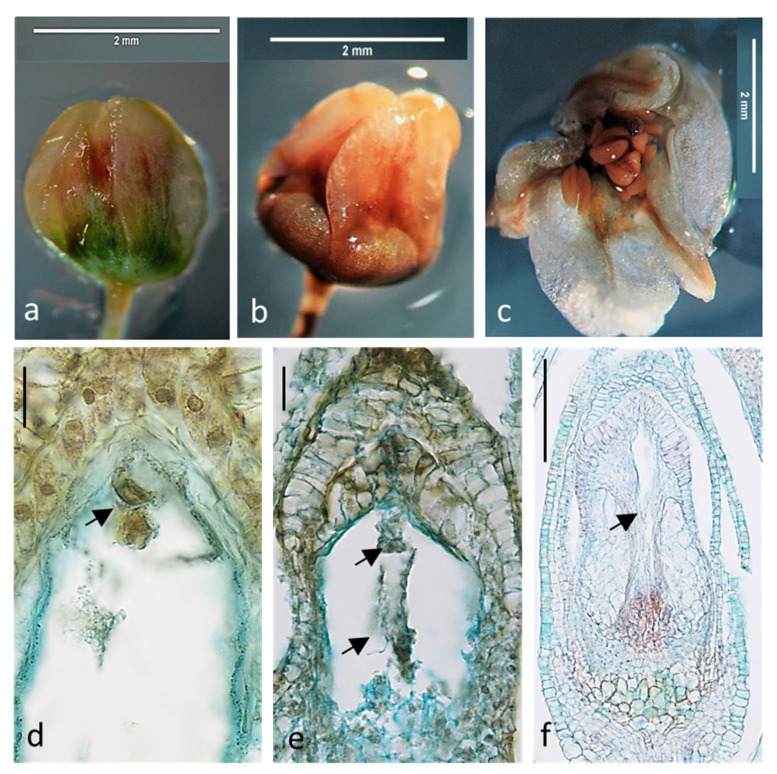
*Fagopyrum esculentum* (cv. ‘Korona’) floral buds and their internal ovule and embryo sac development impairment cultured in vitro on media with different content of sugar, vitamins, and macro- and microelements. The same features were observed in cv. ‘Panda’: (**a**) bud laid out on Medium 1 with full content of nutrients at the time 0; (**b**) bud after 10 days of culture on Medium 1 with 30% content of nutrients; (**c**) bud after 10 days of culture on Medium 1 with full content of nutrients; (**d**,**e**) degeneration of the cells of embryo sacs (arrows); and (**e**) shrunken embryo sac (arrow). Bars: (**d**,**e**) 20 µm; and (**f**) 200 µm. For media content, see Section 4.

**Figure 2:**
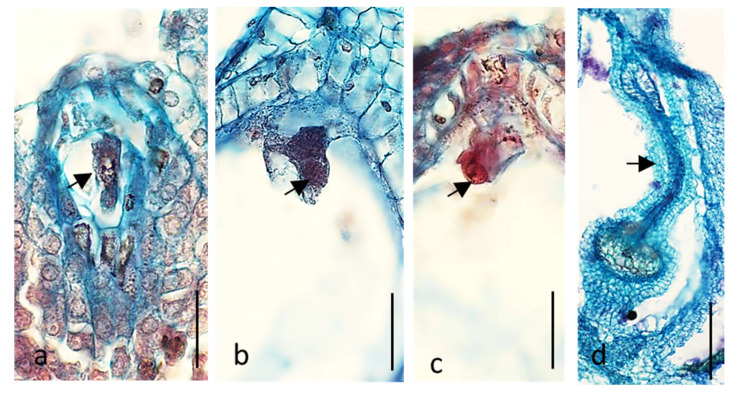
In planta degeneration of the embryo sacs cells and ovules after removal of 75% of flowers in *Fagopyrum esculentum* (cv. ‘Korona’). The same features were observed in cv. ‘Panda’: (**a**) 1-nucleate embryo sac (arrow); (**b**,**c**) egg apparatus of seven-cell embryo sacs (arrows); and (**d**) the whole ovule (arrow). Bars (**a**–**c**) 20 µm; and (**d**) 100 µm.

**Figure 3:**
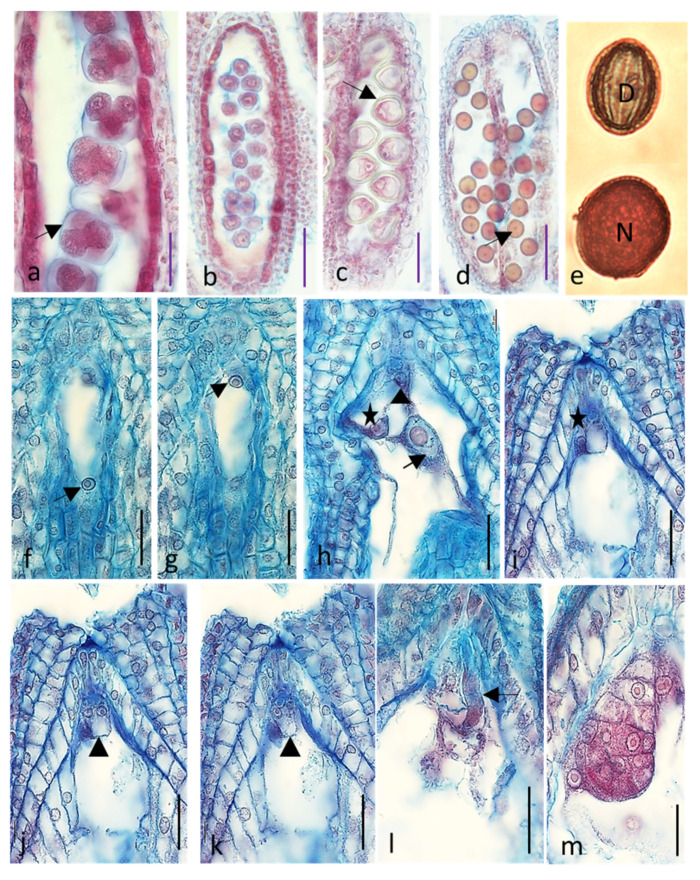
Normal pollen, female gametophyte and embryo development in *Fagopyrum esculentum* (cv. ‘Korona’) after removal of 50% of flowers. The same features were observed in cv. ‘Panda’: (**a**) tetrads of microspores in blue callose sheath (arrow); (**b**) microspores released from the callose sheath; (**c**) vacuolated microspores with thick sporodermis (arrow); (**d**) 1-nucleate pollen grains, nuclei visible (arrow); (**e**) degenerated (D) and normal (N) pollen grains stained with Alexander dye; (**f**,**g**) 2-nucleate embryo sac-successive stages, nuclei marked with arrows; (**h**–**k**) cells of two seven-cell embryo sacs (antipodal cells not shown) with secondary nucleus (arrow), egg cell (stars), and synergids (triangles); (**i**–**k**) successive stages of the same embryo sac; (**l**) pollen tube penetrating one of the two synergids (arrow); and (**m**) globular proembryo. Bars: (**a**,**f**–**m**) 20 µm; (**b**,**c**) 50 µm; and (**d**) 100 µm.

It was evident that in most cases floral buds of both cultivars kept on media with reduced content of sugar, vitamins, and macro- and microelements showed deterioration in growth and development of ovules and embryo sacs. The cells of embryo sac degenerated, and the ovules narrowed (Table 1 and Figure 1). In the case of cv. ‘Panda’ on Medium 1, only drastic reduction of nutrients increased dramatically the percentage of degenerated embryo sacs as compared with that of the control. On Medium 2, there was no effect of nutrients content reduction and on Medium 3 even an increase of the number properly developing ovules was noticed. The development of ovules and embryo sacs was much worse in full content Medium 1 in ‘Korona’ than in ‘Panda’, and further (caused by a reduction in the content of ingredients) deterioration of the ovules quality, although statistically significant, was not as drastic as in Panda. On Media 2 and 3, 50% reduction in nutrients increased the number of defective embryo sacs in ‘Korona’ cv. These findings from in vitro studies supported further in planta experiments. In vitro nutrients’ content reduction decreased the number of properly developed ovules and embryo sacs similarly as in planta depletion of assimilates resulting from flower overproduction in plants without flower removal. They clearly demonstrated that the removal of 75% of flowers had the most negative effect on the frequency of properly formed embryo sacs and ovules (Table 1 and Figure 2). Single-shoot cultivation (1S) also decreased the percentage of properly developed ovules and embryo sacs but to a lesser extent than removing 75% of flowers (Table 1). Removal of 50% of flowers significantly increased or did not change the frequency of properly developed ovules and embryo sacs or pollen viability (Table 1 and Figure 3). Overall, pollen viability in both cultivars and all treatment was high (Table 1). In 97% of cv. ‘Korona’ and in 85% of cv. ‘Panda’ ovules, normal microgametophytogenesis (Figure 3a–e) and megametophytogenesis (Figure 3f,g) were observed, leading to the formation of seven-cell embryo sac of *Polygonum* type (Figure 3h–l). In some ovules, embryos were also observed (Figure 3m).

### 2.2. Phytohormones

The studied cultivars differed significantly in changes of the level of growth regulators in response to the removal of organs competing for assimilates. The most significant changes were observed in cv. ‘Korona’ (Table 2). In buds of these plants in the main-shoot cultivation (1S), significant increases in IAA (indole-3-acetic acid), CYT (sum of cytokinins), JA (jasmonic acid), SA (salicylic acid), and BA (benzoic acid) were noted as compared with the buds of the control (non-treated) plants. IAA, JA, SA, and BA levels increased in open flowers which were capable of fertilization. The wilted flowers (unattractive for pollinators) were additionally richer in GAs (sum of active giberellins).

Interestingly, cv. ‘Korona’ plants after removal of 50% of flowers demonstrated a significant increase in the number of properly developed embryo sacs described above (Table 1). In these buds, significantly higher levels of IAA, ABA (abscisic acid), JA, SA, and BA were recorded as compared with the control plants, and the amounts of JA, SA, and BA were also much higher than in the buds of plants from the other treatments. In open flowers capable of fertilization in this group of plants, less GAs and CYT and significantly more ABA, JA, SA, and BA were detected than in the control plants (Table 2), with particularly growth of SA and BA. Similar differences between control and removal of 50% of flowers in SA and BA content were observed in wilted flowers. Additionally, in these flowers, higher content of IAA was detected. The ratio of GAs to ABA in plants from which 50% of flowers were removed was 1.2 in floralbuds, 0.82 in open flowers, and 1.68 in wilted flowers. On the other hand, in control plants of cv. ‘Korona’, this ratio was much higher (3.8, 1.8, and 2.1, respectively), proving a significant predominance of gibberellins over ABA.

In the buds of cv. ‘Korona’ plants, from which 75% of the flowers were removed, the most significant increase was detected for IAA and ABA. The open flowers demonstrated the highest decrease in IAA and JA and an increase in GAs. The wilted flowers also accumulated less IAA and CYT and more GAs than the control ones (Table 2).

In cv. ‘Panda’, the changes in hormonal profile in flowers under removal were slight (Table 3). In almost all treatments, the greatest fluctuations occurred in the amount of BA, which is a precursor of SA. Increase in SA was recorded in open and wilted flowers of plants from which 75% of flowers were removed. Additionally, in wilted flowers of all treatments, a rise in GAs content was noted (Table 3).

### 2.3. Yield

Statistical analysis showed that the number of flowers produced by the plant was influenced by the cultivation method, i.e., treatment by removing part of the flowers or all lateral ramifications (Table 4). The number of mature seeds and their weight depended on the genotype and treatment. The percentage of flower and fruit (seed) abortion depended only on the genotype, while the efficiency of seed setting was influenced by the interaction of both factors (Table 4).

**Figure 4:**
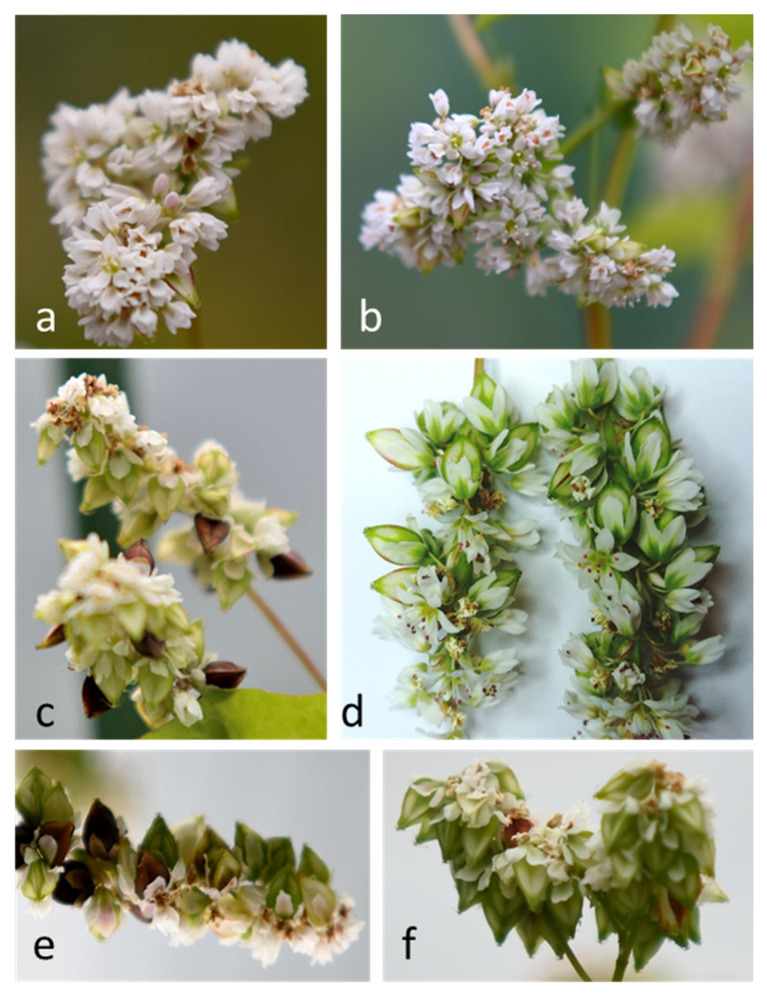
Macroscopic images of flower production (**a**,**b**) and seed setting (**c**–**f**) in cv. ‘Panda’ (**a**–**d**) and cv. ‘Korona’ (**e**,**f**) of *Fagopyrum esculentum*. Please compare control plants in bloom with plants from which 50% of flowers were removed:(**a**) vs. (**b**) and control plants in fruiting with plants with single shoot plants: (**c**) vs. (**d**,**e**) vs. (**f**).

Removing 50% of flowers increased their production in cv. ‘Panda’ (Figure 4a,b and Table 5). This effect was visible in general but particularly in individual cases when the number of flowers even doubled or tripled in relation to the control. This effect was not noted in cv. ‘Korona’. Removing 75% of flowers and single-shoot cultivation in both cultivars significantly reduced the number of flowers produced by one plant as compared with that of the control (Table 5). However, the number of flowers did not affect the number of mature seeds. Only when 50% or 75% of flowers were removed, cv. ‘Panda’ increased the number of seeds. ‘Panda’ and ‘Korona’ plants with one main shoot left produced the lowest number of mature and empty seeds in comparison with those of the control; however, in cv. ‘Korona’, the mass of a single seed was the highest and amounted to 0.0334 g. Smaller number of mature seeds translated into better seed filling and consequently increased mass of thousand seeds (Table 5). Plants in single-shoot cultivation finished vegetation earlier and their seeds matured more quickly than the control plants and plants with partial removal of flowers (Figure 4c–f). Generally, cv. ‘Korona’ showed significantly greater mass of single seed than cv. ‘Panda’ (Table 5). No correlation was found between the number of empty seeds and the number of flowers; however, a positive correlation was found between flower production and abortion of flowers and fruits (*r* = 0.62; *p* < 0.05). The percentage of empty seeds did not correlate with the number of mature seeds, but it correlated negatively with the mass of a single mature seed (*r* = −0.857; *p* < 0.05). Flower production also had an impact on seed setting efficiency—the fewer flowers the plant produced, the more seeds were set. The strongest effect was observed for cv. ‘Panda’, in which the removal of 75% of flowers doubled the seed setting efficiency. The correlation between flower and fruit abortion and seed setting efficiency was very high (*r* = −0.998; *p* < 0.05), similarly as between the number of flowers per plant and seed setting efficiency (*r* = −0.833; *p* < 0.05). The removal of 50% of flowers increased the mass of seeds collected per plant, and MTS increased in cv. ‘Panda’, while, in cv. ‘Korona’, it was similar to that of the control and plants in the remaining treatments (Table 5).

## 3. Discussion

Due to the challenges of common buckwheat cultivation described in the Introduction, especially regarding its low yield, breeding and genetic studies have been carried out for decades (for review, see Matsui and Yasui [16]). Genomic Selection in Mass Selection Breeding program for common buckwheat is a powerful program enhancing buckwheat yield by almost 21% [11]. However, classical breeding treatments such as the one described in this study shed a light on the mechanisms involved in seed production of *F. esculentum*. Key genes involved in seed development are already recognized, and they are genes responsible for Ca^2+^ signal transduction pathway, hormone signal transduction pathways, and coding transcription factors (TFs), as well as starch biosynthesis-related genes [17]. Regarding seed size, AP2 and bZIP transcription factors, BR-signal, and ABA are considered the most important regulators [18].

Our in vitro experiment showed that depriving flowers of nutrients leads to deterioration of their quality and to the abortion of ovule sacs, therefore we decided to start reducing the number of inflorescences in common buckwheat. We were also inspired for the further studies by the never-ending flower overproduction of common buckwheat throughout the whole season.

In our in planta experiment, we partly followed the suggestions of Guglielmini et al. [10], not knowing their outcome while conducting our study in 2019. They recommended to determine the causes of reduction in the number of achenes per raceme (= fruits per inflorescence) during the critical period (the period when grain number is determined and it is crucial to obtain higher yields), as they showed that radiation restriction and subsequent assimilate limitation could increase floret mortality and thereby the number of achenes per raceme.

Yabe et al. [11], who used 92FE1-F4, a population produced by bulk crossing of ‘Tempest’, ‘Kitawasesoba’, ‘Natsusoba’, and ‘Shinanonatsusoba’ cultivars, showed that the number of clusters positively correlated with seed yield. We did not find such a correlation in any of the investigated cultivars. On the contrary, we demonstrated a positive correlation between flower production and the abortion of flowers and fruits.

‘Korona’ plants after removal of 50% of flowers showed significantly lower number of degenerated embryo sacs and higher number of mature seeds, higher efficiency of seed setting than the control plants and plants from the other variants. Since we detected more IAA in these flowers, it is possible that the auxin supports development of embryos, which could be important for higher seed yield in these plants. Although only 3% of degenerated embryo sacs were found, the percentage of aborted flowers did not drop. This may be due to limited pollination or impaired embryo development, which in cv. ‘Panda’ flowers were at 9% and 13% depending on the type of flower (Pin or Thrum) [4]. Despite higher percentage of abnormally formed embryo sacs in cv. ‘Korona’, higher effectiveness of seed setting, calculated as the number of seeds divided by the number of flowers, was found after removing 75% of flowers vs. 50% of flowers. ‘Panda’ plants with 75% flowers removed also showed a much higher percentage of degenerated embryo sacs than the plants treated differently, but the seed setting efficiency was similar to plants with 50% of flowers removed. It is possible that this effect was caused by a higher concentration of gibberellins and that these hormones are more effective than auxin in keeping the embryos alive, similarly as in *Arabidopsis thaliana* [19].

Salicylic acid plays a crucial role in flowering and luring insects, similarly to jasmonic acid. Moreover, both hormones are involved in defense responses of plants in the event of a pathogen attack [20]. Jasmonic acid is also necessary for the formation of an ovulum, and its absence is characteristic of sterile flowers [21]. Such hormones as gibberellins, brassinosteroids, and abscisic acid are of great importance during flowering. It is usually believed that ABA, as an antagonist of gibberellins, inhibits flowering [22]. It was therefore unexpected that the flowers that set seeds the most efficiently accumulated higher levels of this hormone than those of plants with a greater degree of embryo sac degeneration. GA to ABA ratio in plants from which 50% of flowers were removed was much lower than in the control plants. These results mean that the proportion between gibberellins and abscisic acid is more important than absolute concentrations of these hormones. On the other hand, in the flowers of control plants, this ratio was much higher, proving a significant predominance of gibberellins over ABA, and at the same time these plants were characterized by a greater degree of ovule sac degeneration. These findings thus indicate that a direct reason for embryo sac abnormal development is probably independent of these two classes of hormones.

Salicylic acid controls, similar to JA, the process of attracting insects during the flowering phase. However, while JA confers flowers their attractive fragrance, SA can increase flower temperature to release volatile compounds [23]. Benzoic acid (BA) is a precursor of salicylic acid, so it was not surprising that BA concentration in flowers was high and translated directly into high content of SA. Our results indicate that the two cultivars studied also differed in terms of producing the hormones responsible for luring insects. ‘Panda’ flowers accumulated much greater amounts of salicylic acid, while ‘Korona’ ones produced mainly jasmonic acid. It should also be remembered that majority of hormones stimulate flower formation in the vegetative phase, so the presence of hormones in flowers may not give a complete picture of the role of individual hormones in embryological development.

Taylor and Obendorf [12] argued that poor seed yielding in common buckwheat results from problems with ovule development and fertilization. They also claimed that the lack of fertilization is influenced by variable viability and quality of microspores. Our research shows that pollen viability in both studied genotypes is high, and even enhanced by flower removal in cv. ‘Panda’. This research further confirmed our long-term observations of a strong positive correlation between the number of flowers and their abortion, which means that the more flowers a plant produces, the more of them are rejected. According to ourresults, flower abortion is primarily influenced by plant genotype, and this barrier would be very difficult to break. In our opinion, buckwheat plants have a certain limit of seeds, and, above this limit, the plant will not allocate its ‘resources’ (assimilates) to their filling. A very high negative correlation between flower abortion and efficiency of seed setting was found, which proves that a decrease in assimilate competition significantly increases seed setting. As we noticed, the plant seems to be reaching a certain limit of flower production, and it is difficult to change this limit. Halbrecq et al. [13] reported that the abortion mainly affects flowers from the upper floors, that is, those arising in the later stage of flowering, in relation to the flowers located lower, i.e., previously produced. Sugawara [24] and Asako et al. [25] found that flowers formed earlier are more likely to set seeds than flowers formed later. Our previous unpublished research showed that the nectar is richer in sugars in earlier flowers than in later ones. This factor likely has a significant impact on the flight of pollinating insects, which directly translates into seed setting. In addition, Taylor and Obendorf [12] reported that the embryo sacs are better developed in the earlier formed flowers. Our observations indicate that flower abortion occurs throughout the flowering period and its degree depends on many factors. In buckwheat, the period of vegetative development overlaps with the period of generative organ formation. The formation of vegetative organs coincides with intense flowering and seed formation, which results in strong competition for assimilates. According to Halbrecq et al. [13], when plants reach their maximum vegetative development, competition for assimilates decreases. Assimilates can therefore be located mainly in the generative organs. These authors claimed that more flowers and seeds are produced during this time, and the degree of abortion decreases along with competition for assimilates. Our research did not confirm these conclusions. We proved that the increased number of flowers did not correlate with the number of seeds, because at the same time the abortion of flowers increased. In previous studies, we observed a certain percentage of ‘starved’ embryos, so the competition for assimilates between the set seeds and the still emerging flowers continued [4]. The present experiments confirmed a negative correlation between the number of empty seeds and mass of a single seed.

Halbrecq et al. [13] performed an experiment involving defoliation (partial and total) and partial removal of buckwheat inflorescences from the main shoot in order to modify the availability of assimilates and reduce competition between seeds. In all cases, regardless of the procedure, they observed a drastic reduction in the number of grains in relation to the number of flowers formed, with very low seed yield, around 20–30%. The authors found that the critical seed setting stage occurs shortly after flowering and is not affected by a change in donor-acceptor relationship due to defoliation or removal of part of the inflorescences. The drastic limitation of competition between inflorescences and seeds had only a negligible effect on the final grain yield per plant, which indicated a strong compensation by the remaining grains. In our research, we showed that only the removal of 50% of flowers allowed for increasing the seed yield. In the remaining cases, removing flowers reduced the yield or did not change it as compared with the control. Despite the fact that the percentage of empty seeds did not significantly correlate with the number of whole seeds, it significantly negatively correlated with the weight of individual mature seed. These findings clearly indicate that the setting of seeds, although not filled later on, reduced the mass of mature seeds. It can therefore be assumed that flowering and fertilization are the phases critical for seed yield. Flower overproduction and embryo formation is a process that exhausts the plant reserves. Failure to fill all the seeds will no longer compensate for these losses. Forming empty seeds is therefore, apart from flower abortion, another form of crop regulation when the plant produced too many flowers.

It is worth underlining that plants cultivated as single-shoots finished their blooming period earlier and their seeds matured also earlier than those of the other plants. The study results confirm our hypothesis that plants with a shorter flowering period (self-finishing) may achieve higher seed yield. Considering that the number of flowers, their abortion, and the percent of defective embryos are controlled genetically, breeders are faced with a challenging task of producing new genotypes with amended traits. Other difficulties involve strong self-incompatibility and impossibility of inbreeding. Given these limitations, mutations seem to be the only way to obtain new forms of common buckwheat, and we will explore this approach in the years to come.

## 4. Materials and Methods

### 4.1. In Vitro and In Planta Experimental Design

Investigation of trophic (nutrient) stress in in vitro conditions was performed in 2019, while the experiments conducted in planta were performed twice in 2019 and 2020. The presented results are the means of two independent experiments. The seeds of *F. esculentum* of Polish cultivars ‘Panda’ and ‘Korona’ used for the experiment were provided by Małopolska Hodowla Roślin in Polanowice Sp. z o.o. (Poland).

#### 4.1.1. In Vitro Experiment

Plants obtained from the seeds were cultivated in pots filled with a commercial soil substrate (pH = 6.0) mixed with perlite 1:1 (*v*:*v*) in a phytotronic chamber at 20 °C and humidity of 50–60%, under 16 h photoperiod and 300 μmol m^−2^ s^−1^ of PPFD (photosynthetic photon flux density). Large buds 2.25–3.50 mm in size were collected from two-month-old plants during full blossom stage and sterilized in 70% ethanol for 1 min and 20% sodium hypochlorite for 7 min. Then, they were washed three times in sterile water for 5 min. After sterilization the buds were transferred onto three different media, containing 100%, 50%, and 30% of sugar, vitamins, and macro- and microelements. The full content media was prepared according to the in vitro cultivation protocol of common buckwheat: (1) ½ MS (Murshige & Skoog) + 30 g dm^−1^ sucrose + BA 1 mg dm^−1^ + NAA 0.1 mg dm^–1^ [25]; (2) MS + vit. B_5_ 2 mg dm^−1^ + sucrose 25 g dm^−1^ + glutamine 0.1 g dm^−1^ + kinetin 1 mg dm^−1^ + GA_3_ 1 mg dm^−1^ [1]; and (3) MS + 30 g dm^−1^ sucrose + zeatin 2 mg dm^−1^ [25]. pH of the media was established at 5.6, and 0.8% agar was used for solidification. All compounds were obtained from Sigma-Aldrich (St. Louis, MO, USA). The lowest content (30%) of the medium ingredients was used only in Medium 1. The floral buds extracted from inflorescences were cultivated on the media for ten days at a constant temperature of 20 ± 2 °C, relative humidity of air 50–60%, 16 h photoperiod, and 300 μmol m^−2^ s^−1^ PPFD (AGRO Philips sodium lamps, Philips, Aachen, Germany), and then collected for embryological analyses.

#### 4.1.2. In Planta Experiment

**Figure 5:**
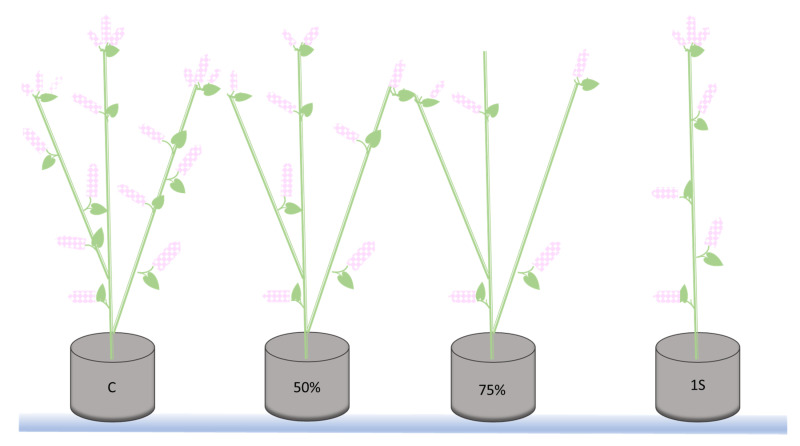
Inflorescences and lateral stem removal performed during in planta experiments on *Fagopyrum esculentum* in 2019 and 2020. C, control plant with all lateral ramifications and with all inflorescences; 50%, plant with half of the spike of spikelets (every second removed); 75%, plant with only 25% of spike of spikelets (every second, third, and fourth removed); 1S, single shoot plant (all lateral ramifications removed).

In 2019 and 2020, the influence of in planta assimilate availability on the embryological development and yielding of buckwheat plants was investigated. Plants obtained from the seeds were grown from May to September in pots filled with commercial soil substrate (pH = 6.0) mixed 1:1 (*v*:*v*) with perlite in an open tunnel enabling the flight of insects. The plants were divided into four groups, control (without flower removal), single main-shoot cultivation (successive removal of all inflorescence developing on lateral branches and lateral branches as well, marked 1S), 50% (every second inflorescence, i.e., spike of spikelets, was removed), or 75% (every second, third, and fourth spike of spikelets was removed), the latter two marked 50% or 75%, respectively (Figure 5). The flowers for embryological and hormonal analyses were collected during full flowering phase.

### 4.2. Embryological Processes in Flowers in In Vitro and In Planta Experiments

After 10 days of the bud culture on artificial media and at the time of flower number counting (mid-July), enlarged buds (in vitro) and buds and open flowers left on the plants (in planta experiment from 2019) were fixed in a mixture of acetic acid and 96% ethanol (1:3; *v*:*v*) for 24 h at room temperature. Fixed flowers were kept in 70% ethanol for further analyses. Dehydration, paraffin supersaturation, embedding, and slicing were conducted as described in detail for *F. esculentum* by Płażek et al. [6]. Staining was performed as described by Słomka et al. [4]. From 20 to 30 flowers per treatment were analyzed.

Pollen viability (stainability) in the flowers from in planta experiment from 2019 was assessed in 2200–3500 pollen grains per treatment (from at least 20 flowers) by staining with Alexander dye [26]. Isolated pollen grains were stained on a microscopic slide and the number of viable (purple) and non-viable (green or transparent) pollen grains was counted.

### 4.3. Phytohormonal Profile Analyses in Flowers in In Planta Experiments

Analysis of selected plant hormones was performed as reported previously [6,27,28,29]. Lyophilized and pulverized plant material (15 mg) was triple extracted in 1 mL of methanol/water/formic acid (15:4:1; *v*:*v*:*v*) solution. At this stage, a heavy-labeled internal standards mixture was added (about 20 pmol of [^15^N_4_]dihydrozeatin, [^2^H_5_]*trans*-zeatin-riboside, [^15^N_4_]kinetin, [^2^H_2_]gibberellin A_1_, [^2^H_2_]gibberellin A_4_, [^2^H_2_]gibberellin A_6_, [^2^H_2_]gibberellin A_5_ [^2^H_5_]indole-3-acetic acid, and [^2^H_6_]*cis,trans*-abscisic acid, [^2^H_4_]salicylic acid, [^2^H_5_]benzoic acid (OlChemim, Olomouc, Czech Republic), and [^2^H_5_]jasmonic (CND Isotopes, Quebec, QC, Canada)). Samples after evaporation (N_2_ stream) were reconstituted (3% methanol in 1 M formic acid) and cleaned up on hybrid SPE columns (BondElut Plexa PCX, Agilent, Santa Clara, CA, USA). Measurements were conducted on ultrahigh performance liquid chromatograph (UHPLC, Agilent Infinity 1260) coupled to 6410 Triple Quad LC/MS with ESI (Electrospray Interface) ion source (Agilent Technologies) in MRM mode. Technical details are provided in references cited. Quantification was based on calibration curves for authentic standards considering recoveries of heavy-labeled internal standards.

The following compounds were determined: indole-3-acetic acid (IAA), kinetin (KIN), gibberellins (GA_1_, GA_3_, GA_4_, GA_5_, GA_6_, and GA_7_), *cis, trans*-abscisic acid (ABA), salicylic acid (SA), benzoic acid (BA), and jasmonic acid (JA). Five plants per treatment were analyzed.

### 4.4. Yield Related Measurements in In Planta Experiments

The number of flowers produced by control, 1S, 50%, and 75% plants were assessed in mid-July 2019 and 2020 in 20 replicates (20 plants) from each treatment. The seeds were harvested at the end of August 2019 and 2020. The number of mature and empty seeds, as well as the mass of mature seeds per plant were counted as a mean of 20 plants per treatment. The mass of one thousand seeds (MTS) was also calculated. The percentage of flower and embryo abortion was calculated according to the formula: (1 − mature seed number/number of flowers) × 100%. The efficiency of seed setting was calculated according to the formula: (mature seed number/number of flowers) × 100%.

### 4.5. Statistical Analysis

All results from in planta experiments were analyzed by ANOVA. Differences between means were calculated using Duncan’s multiple range test (*p* < 0.05). The values show the means ± SE (standard error). Correlations between the studied parameters (Pearson’s coefficient) were tested at *p* < 0.05. In the case of not normal distribution, the non-parametric Chi-square test was used. The Chi-square test of independence was performed for the frequency of ovule and embryo sac disturbances. All statistical analyses were performed in Statistica v. 13 (Statsoft, Kraków, Poland).

## 5. Conclusions

The conclusions and postulated mechanisms of seed yield regulation in buckwheat plants are as follows:

1. Following the experimental loss of some flowers, a plant initiates compensation processes including: increase in the efficiency of pollen viability by reducing the percentage of degenerated pollen grains; production of additional flowers, but, when the number of flowers turns out to be too high, the plant aborts most of them; increase the percentage of empty seeds if the above-mentioned measures are insufficient; and reduction in the amount of reserve materials accumulated in the seeds, and thereby reduction of seed mass if the other mechanisms prove insufficient.
2. The critical point for seed yield is the moment of flowering and fertilization. Flower overproduction and embryo formation are the processes that exhaust the plant reserves. Failure to fill all the seeds will no longer compensate for these losses. Forming empty seeds is therefore, next to flower abortion, another form of crop regulation when the plant has produced too many flowers. Our study confirmed the common observation that the greater is the number of seeds, the smaller is the mass of a single seed.
3. Removing 50% of flowers significantly reduces the percentage of defective embryo sacs, which has a direct impact on increasing the yield of mature seeds. However, this relationship was only observed in cv. ‘Korona’. In this cultivar, this effect can be attributed to higher concentration of jasmonic acid, salicylic acid (and its precursor - benzoic acid), which play an important role in attraction of pollinators.
4. Plants in single-shoot cultivation finish their vegetation earlier and achieve higher mass of one seed as compared with that of the control. This result confirms our hypothesis that self-finishing plants of common buckwheat, with shorter blooming phase, could deliver higher seed yield.

## Figures and Tables

**Table 1:** The influence of medium content on in vitro cultured floral buds, and of flower removal on embryological parameters in cv. ‘Panda’ and ‘Korona’ of *Fagopyrum esculentum*.

In Vitro					
Medium	Content of Compounds (%)	Frequency (%) of Degenerating Embryo Sacs and Ovules			
		‘Panda’ cv.		‘Korona’ cv.	
1	100	0		38	
	50	0		42 *	
	30	68 *		50 *	
2	100	15		36	
	50	14		50 *	
3	100	29		13	
	50	0 *		38 *	
**In Planta**					
**Treatment**		**‘Panda’ cv.**		**‘Korona’ cv.**	
		**Frequency (%) of Degenerated**			
		**Pollen Grains**	**Embryo Sacs and Ovules**	**Pollen Grains**	**Embryo Sacs and Ovules**
Control		3.2	10	1.3	23
1S		3.1	50 *	1.6	40 *
50%		2.2 *	15	0.9	3 *
75%		1.1*	66 *	1.2	85 *

Chi-square test (*p* < 0.05) was performed separately for in vitro and in planta treatments. Means marked with asterisks (*) differ significantly from control. In vitro: Analysis performed on 25–30 ovules/treatment. For media content, see Section 4. Media with 100% composition were controls. In planta: Control, plants with all flowers and lateral ramifications; 1S, plants with only one main shoot; 50% and 75%, percentages of flowers removed.

**Table 2:** Phytohormone content (µmol g^−1^ DW) in floral, and open and wilted flowers of common buckwheat plants of cv. ‘Korona’ cultivated with only one main shoot (1S) or after removal of 50% or 75% of flowers. Control, plants with all lateral ramifications and flowers. Analyses were done in the phase of full blooming.

Hormones	Buds				Open Flowers				Wilted Flowers			
	Control	1S	50%	75%	Control	1S	50%	75%	Control	1S	50%	75%
IAA	73.5 ± 7.1 ^d^	124 ± 11 ^b^	102 ± 10 ^c^	149 ± 13 ^a^	67.9 ± 6.2 ^b^	81.7 ± 8.0 ^a^	64.3 ± 6.1 ^b^	47.2 ± 4.3 ^c^	157 ± 15 ^c^	189 ± 17 ^b^	253 ± 21 ^a^	66.1 ± 6.1 ^d^
GAs	8.29 ± 0.79 ^a^	8.14 ± 0.72 ^a^	3.51 ± 0.30 ^b^	8.50 ± 0.81 ^a^	7.40 ± 0.72 ^a,b^	6.45 ± 0.61 ^b^	4.59 ± 0.47 ^c^	8.67 ± 0.81 ^a^	7.40 ± 0.73 ^c^	11.1 ± 0.11 ^b^	6.87 ± 0.65 ^c^	15.1 ± 0.12 ^a^
CYT	2.88 ± 0.25 ^c^	8.93 ± 0.83 ^a^	2.55 ± 0.25 ^c^	3.91 ± 0.36 ^b^	3.78 ± 0.35 ^a^	3.92 ± 0.32 ^a^	2.10 ± 0.19 ^b^	3.36 ± 0.32 ^a^	4.09 ± 0.37 ^a^	4.43 ± 0.42 ^a^	4.49 ± 0.41 ^a^	2.40 ± 0.21 ^b^
ABA	2.16 ± 0.19 ^c^	1.55 ± 0.12 ^d^	2.92 ± 0.25 ^b^	3.50 ± 0.31 ^a^	4.04 ± 0.36 ^b^	2.69 ± 0.25 ^d^	5.63 ± 0.52 ^a^	3.53 ± 0.29 ^c^	3.45 ± 0.33 ^b^	1.80 ± 0.17 ^d^	4.09 ± 0.39 ^a^	2.52 ± 0.26 ^c^
JA	51.42 ± 5.07 ^c^	94.61 ± 9.11 ^b^	151 ± 14 ^a^	30.82 ± 3.98 ^d^	24.12 ± 2.95 ^c^	54.14 ± 5.12 ^a^	30.71 ± 2.76 ^b^	6.46 ± 0.68 ^d^	34.94 ± 3.32 ^b^	56.22 ± 5.21 ^a^	58.82 ± 5.76 ^a^	59.15 ± 5.78 ^a^
SA	22.6 ± 2.1 ^c^	77.8 ± 7.2 ^a^	86.8 ± 8.3 ^a^	41.9 ± 3.9 ^c^	18.8 ± 1.8 ^c^	62.4 ± 6.1 ^b^	105 ± 9.5 ^a^	65.1 ± 5.8 ^b^	33.2 ± 3.2 ^c^	96.7 ± 9.3 ^b^	124 ± 10.2 ^a^	31.7 ± 2.8 ^c^
BA *	31.3 ± 2.8 ^b^	68.4 ± 5.5 ^a^	62.7 ± 5.2 ^a^	28.6 ± 1.9 ^b^	21.2 ± 1.6 ^c^	33.2 ± 2.8 ^b^	41.8 ± 3.8 ^a^	27.6 ± 2.4 ^c^	16.2 ± 1.1 ^c^	25.9 ± 2.3 ^b^	38.4 ± 2.9 ^a^	36.4 ± 1.8 ^a^

Values represent means (*n* = 5 in each experiment) of two experiments performed during 2019–2020 ± SE (standard error). Different superscript letters (a–d) within rows for each hormone and organ indicate significant differences between means (Duncan’s multiple range test; *p* < 0.05). IAA, indole-3-acetic acid; active GAs, sum of active gibberellins (GA_1_, GA_3_, GA_4_, GA_5_, GA_6_, and GA_7_); CYT, sum of cytokinins (kinetin, zeatin, *N*6-izopentenyladenine, and *N*6-izopentenyladenozine); ABA, abscisic acid; JA, jasmonic acid; SA, salicylic acid; * BA, benzoic acid, a precursor of salicylic acid.

**Table 3:** Phytohormone content (µmol g^−1^ DW) in buds, open and wilted flowers of common buckwheat plants of cv. ‘Panda’ cultivated with only one main shoot (1S) and after removal of 50% or 75% of flowers. Control, plants with all lateral ramifications and flowers. Analyses were done in the phase of full blooming.

Hormones	Buds				Open Flowers				Wilted Flowers			
	Control	1S	50%	75%	Control	1S	50%	75%	Control	1S	50%	75%
IAA	173 ± 18 ^a^	77 ± 6 ^c^	110 ± 10 ^b^	110 ± 9 ^b^	91 ± 8 ^a^	73 ± 7 ^b^	69 ± 6 ^b^	71 ± 6 ^b^	287 ± 26 ^a^	267 ± 24 ^a^	133 ± 12 ^b^	134 ± 12 ^b^
GAs	5.84 ± 0.42 ^a^	4.47 ± 0.40 ^b^	6.22 ± 0.52 ^a^	4.63 ± 0.39 ^b^	6.22 ± 0.59 ^a^	6.87 ± 0.58 ^a^	5.65 ± 0.57 ^a^	5.60 ± 0.48 ^a^	6.15 ± 0.57 ^c^	10.85 ± 0.99 ^a^	8.67 ± 0.82 ^b^	10.26 ± 0.11 ^a^
CYT	3.58 ± 0.31 ^b^	2.53 ± 0.21 ^c^	5.09 ± 0.45 ^a^	3.29 ± 0.31 ^b^	3.72 ± 0.34 ^a,b^	3.46 ± 0.32 ^b^	4.16 ± 0.41 ^a^	3.83 ± 0.32 ^a^	3.22 ± 0.35 ^b^	4.39 ± 0.38 ^a^	5.01 ± 0.46 ^a^	3.27 ± 0.32 ^b^
ABA	2.74 ± 0.21 ^a^	1.88 ± 0.19 ^b^	1.51 ± 0.14 ^b^	1.30 ± 0.14 ^c^	5.36 ± 0.49 ^a^	3.99 ± 0.32 ^b^	3.75 ± 0.33 ^b^	2.78 ± 0.27 ^c^	3.31 ± 0.29 ^a^	2.54 ± 0.26 ^b^	1.92 ± 0.17 ^c^	3.43 ± 0.34 ^a^
JA	80.42 ± 7.01 ^a^	80.40 ± 0.76 ^a^	34.61 ± 3.20 ^c^	53.36 ± 5.01 ^b^	14.43 ± 1.42 ^b^	20.37 ± 2.04 ^a^	9.67 ± 0.91 ^c^	9.04 ± 0.90 ^c^	46.91 ± 4.05 ^a^	48.99 ± 4.12 ^a^	15.50 ± 1.40 ^c^	25.53 ± 2.61 ^b^
SA	56.93 ± 6.02 ^a^	25.62 ± 2.56 ^c^	33.07 ± 3.01 ^b^	59.37 ± 6.02 ^a^	44.59 ± 4.8 ^b^	40.49 ± 4.06 ^b^	40.08 ± 3.89 ^b^	71.24 ± 7.05 ^a^	65.29 ± 6.21 ^b^	48.02 ± 4.78 ^c^	45.0 ± 4.31 ^c^	139 ± 12 ^a^
BA *	29.80 ± 2.01 ^c^	66.00 ± 6.26 ^a^	43.10 ± 4.11 ^b^	45.31 ± 4.23 ^b^	21.55 ± 2.01 ^b^	27.43 ± 2.36 ^a^	30.05 ± 3.01 ^a^	26.90 ± 2.48 ^a^	24.68 ± 2.48 ^b^	36.72 ± 3.76 ^a^	34.91 ± 3.02 ^a^	22.43 ± 2.01 ^b^

Values represent means (*n* = 5 in each experiment) of two experiments performed during 2019–2020 ± SE (standard error). Different superscript letters (a–d) within rows for each hormone and organ indicate significant differences between means (Duncan’s multiple range test; *p* < 0.05). * Benzoic acid is not a hormone but a precursor of salicylic acid. IAA, indole-3-acetic acid; active GAs, sum of active gibberellins (GA_1_, GA_3_, GA_4_, GA_5_, GA_6_, and GA_7_); CYT, sum of cytokinins (kinetin, zeatin, *N*6-izopentenyladenine, and *N*6-izopentenyladenozine); ABA, abscisic acid; JA, jasmonic acid; SA, salicylic acid; * BA, benzoic acid, a precursor of salicylic acid.

**Table 4:** Analysis of variance of the impact of plant treatment (main shoot only, removal of 50% or 75% of flowers, and control) on flowering and fruiting in common buckwheat cv. ‘Panda’ and ‘Korona’. Efficiency of seed setting, seed mass, and mass of thousand seeds (MTS) were calculated per individual.

Effects	No. of Flowers	No. of Mature Seeds	Empty Seeds (%)	Abortion of Flowers and Fruits (%)	Efficiency of Seed Setting	Mature Seed Mass	MTS
Cultivar	ns	***	*	***	ns	***	***
Treatment	***	**	***	ns	ns	***	***
Cultivar x Treatment	ns	***	ns	ns	*	***	***

*, **, and *** indicate statistically significant effect of treatment at *p* < 0.05, *p* < 0.01, and *p* < 0.001, respectively; ns, not significant.

**Table 5:** Effect of removal of all lateral ramifications (1S) or 50% or 75% of flowers on flowering and fruiting parameters of two cultivars of common buckwheat. Control, plants with all flowers and lateral ramifications.

Cultivar	Treatment	No. of Flowers per Plant	No. of Mature Seeds per Plant	No. of Empty Seeds per Plant	Abortion of Flowers and Seeds (%)	Efficiency of Fertilization (%)	Mass of One Seed	Seed Mass per Plant (g)	MTS (g)
‘Panda’	Control	669 ± 55 ^b^	128 ± 9 ^a,b^	29 ± 8 ^a^	81	19	0.0267 ± 0.003 ^e^	3.42 ± 0.07 ^c^	26.72 ± 1.41 ^b^
	50%	823 ± 74 ^a^	136 ± 11 ^a^	25 ± 5 ^a^	84	17	0.0292 ± 0.003 ^d^	3.97 ± 0.09 ^a^	29.19 ± 1.55 ^a^
	75%	321 ± 29 ^e^	130 ± 12 ^a^	26 ± 4 ^a^	59	40	0.0252 ± 0.002 ^f^	3.27 ± 0.08 ^d^	25.15 ± 1.89 ^b^
	1 S	357 ± 32 ^ed^	107 ± 9 ^b^	22 ± 5 ^a^	70	30	0.0266 ± 0.003 ^e^	2.85 ± 0.05 ^f^	26.63 ± 1.99 ^b^
‘Korona’	Control	778 ± 69 ^a^	105 ± 10 ^b,c^	14 ± 2 ^b^	87	13	0.0311 ± 0.003 ^b^	3.27 ± 0.08 ^d^	31.14 ± 2.05 ^a^
	50%	558 ± 52 ^c^	125 ± 11 ^b^	16 ± 4 ^b^	78	22	0.0303 ± 0.002 ^c^	3.79 ± 0.07 ^b^	30.32 ± 2.03 ^a^
	75%	442 ± 45 ^d^	100 ± 8 ^b,c^	22 ± 4 ^a^	77	23	0.0297 ± 0.003 ^c,d^	2.97 ± 0.06 ^e^	29.70 ± 2.07 ^a,b^
	1 S	427 ± 38 ^d^	91 ± 7 ^c^	6 ± 2 ^c^	79	21	0.0334±0.003 ^a^	3.04 ± 0.08 ^e^	33.40 ± 2.27 ^a^

Values represent means (*n* = 20 in each experiment) of two experiments performed during 2019–2020 ± SE (standard error). Different superscript letters (a–f) within columns for each treatment and cultivar indicate significant differences between means (Duncan’s multiple range test; *p* < 0.05). Percentage of flower and embryo abortion was calculated as: (1—No. of mature seeds/No. of flowers) × 100. Efficiency of fertilization was calculated as: (No. of mature seeds/No. of flowers) × 100.

## Abbreviations

ABA	Abscisic acid
BA	Benzoic acid
CYT	Cytokinins
GAs	Active gibberellins (GA_1_, GA_3_, GA_4_, GA_5_, GA_6_, GA_7_)
IAA	Indole-3-acetic acid
JA	Jasmonic acid
SA	Salicylic acid
MTS	Mass of thousand seeds
1S	Plant with single main shoot
50%	Plant with 50% of flowers removed
75%	Plant with 75% of flowers removed
